# Evaluation of Factors Relevant to Pain Control Among Patients After Surgical Treatment

**DOI:** 10.1001/jamanetworkopen.2021.40869

**Published:** 2021-12-28

**Authors:** Natalie B. Baxter, Hoyune E. Cho, Jessica I. Billig, Sandra V. Kotsis, Steven C. Haase, Kevin C. Chung

**Affiliations:** 1 Section of Plastic Surgery, Department of Surgery, Michigan Medicine, Ann Arbor; 2 Department of Plastic Surgery, University of California, Irvine, School of Medicine, Orange

## Abstract

**Importance:**

Opioids are often prescribed after elective surgical treatment despite the potential for misuse. Although various pain control regimens exist, patient preferences for acute postoperative pain management are unknown.

**Objective:**

To describe patient-reported key attributes of postoperative pain management.

**Design, Setting, and Participants:**

This decision analytical model used responses from a survey based on conjoint analysis to investigate the value patients placed on different aspects of postoperative pain management. Participants were patients aged 18 years or older who underwent elective hand surgical procedures between July 1, 2018, and July 23, 2019, at a single academic center. The survey was completed on a web-based platform and took place between November 2019 and January 2020. Data were analyzed from May through July 2021.

**Exposures:**

Participants were presented with a series of discrete-choice tasks and asked to select between 2 postoperative medication options that changed from question to question and had varying characteristics.

**Main Outcomes and Measures:**

Attribute importance scores and part-worth utility values for the queried aspects of pain control were calculated.

**Results:**

Of 710 individuals invited, 321 (45.2%) completed the survey; there were 212 (66.0%) women and 108 (33.6%) men, and the most common age category was 60 to 69 years (102 participants [31.8%]). Most patients reported previous opioid use (282 individuals [87.9%]). Factors in the decision-making process with the highest attribute importance scores (SDs) were risk of addiction (26.3% [13.0%]) and amount of pain relief (25.6% [14.6%]). Adverse effects 13.9% (7.2%), functional independence 11.8% (7.3%), and level of trust in the prescriber 11.4% (5.8%) had intermediate attribute importance scores (SDs). Cost 7.9% (4.4%) and stigma 3.1% (1.3%) had the lowest attribute importance scores (SDs) in patient decisions.

**Conclusions and Relevance:**

These findings suggest that multimodal pain control regimens that are associated with optimized pain relief and minimized risk of addiction are preferable to treat acute postoperative pain. The results suggest that identifying procedures for which patients prioritize minimizing risk of addiction over pain relief and incorporating patient preferences into decision-making may be associated with decreased postoperative opioid prescribing.

## Key Points

**Question:**

Which attributes of pain control are most important to patients after surgical treatment?

**Findings:**

In this decision analytical model study of survey responses from 321 patients who underwent elective hand surgical procedures, risk of addiction and pain relief were the aspects of postoperative pain control with the highest attribute importance scores.

**Meaning:**

These findings suggest that a multimodal pain control regimen that is associated with sufficient pain relief and decreased risk of addiction may be ideal for the treatment of postoperative pain.

## Teaser

This decision analytical model study uses survey responses to investigate the importance patients gave to attributes of postoperative pain management after hand surgical procedures.

## Introduction

Acute postoperative pain is commonly treated with opioids.^1^ However, there is widespread concern regarding opioid misuse and diversion from prescriptions. Therefore, health systems, government organizations, and surgical societies have established guidelines aimed at limiting the number of opioid pills prescribed after surgical treatment.^2^ Despite these efforts, studies have found substantial variation in postoperative opioid prescriptions and an association between opioid prescription and morbidity and mortality.^3,4,5^ It has become apparent that existing opioid reduction policies do not take a patient-centered approach and instead tend to focus on clinician behaviors. A more holistic strategy encompassing patient, clinician, and health system factors is needed to combat the opioid epidemic.

Patient preferences for postoperative pain control are poorly understood and missing from the literature. Little is known regarding the trade-offs patients make for acute pain management, including the positive attributes of pain relief and potential negative consequences of opioid use, such as adverse effects and addiction potential. However, research on management of chronic pain suggests that patients prioritize avoiding opioid-related adverse effects, such as nausea and vomiting, which may also eliminate the net benefits of pain relief associated with opioid use, and that patients are often less concerned with risk of addiction.^6^ For advanced cancer pain in particular, Meads and colleagues^7^ found that patients preferred optimal pain control, low out-of-pocket medication costs, and adequate control of adverse effects. These studies highlight the importance of an individualized approach for chronic pain management. It is unknown whether patients with acute postoperative pain have similar preferences. Given the potential for opioid misuse and poor pain control after surgical treatment, further investigation is warranted.

We sought to use discrete choice experiments (DCEs) to investigate which attributes of pain control, such as risk of addiction and potential for adverse effects, were most important to patients after surgical treatment. DCEs use surveys to evaluate trade-offs and quantify the relative preference that responders have for various attributes of a product or service. Findings from this study may provide insight into patient perspectives on the relative importance of analgesic characteristics and may inform prescription decisions.

## Methods

The Michigan Medicine Institutional Review Board (IRB) determined that this decision analytical model study was exempt from IRB review because we collected the data anonymously (ie, each survey could not be traced back to an individual patient) and separately from the monetary compensation list that contained patient contact information. Informed consent for the voluntary study was obtained using an IRB-approved consent prompt and template (eAppendix in the Supplement). This investigation follows the Consolidated Health Economic Evaluation Reporting Standards (CHEERS) reporting guideline for decision analytical models given that this survey methodology can be used to identify aspects of postoperative pain control that are most important to patients who underwent surgical treatment and had acute pain.

### Participants

We used DataDirect, a tool that provides access to clinical data, to identify patients at Michigan Medicine who underwent outpatient surgical treatment between July 1, 2018, and July 23, 2019. We generated the recruitment list using Common Procedure Terminology codes for common outpatient procedures, such as carpal tunnel release and carpometacarpal joint arthroplasty (eTable 1 in the Supplement). We excluded individuals aged younger than 18 years and those who did not have an email listed in the electronic health record. We sent eligible patients an email with a link to participate in the voluntary survey and offered a $5 mailed check as compensation for completion.

### Survey Methodology

We used conjoint analysis (CA), which is a survey methodology that originated in market economics to determine which elements of a product are most valuable and evaluate the trade-offs consumers consider during product selection.^8,9,10^ CA relies on the theory that people first break a product down into its component attributes and consider which attributes are most desirable and next compare different products while assessing the total value based on component attributes.^8,10^ For example, most people prefer increased fuel efficiency when buying a car, but there is a certain threshold value that a person is willing to accept in exchange for leather-covered seats. CA quantifies this association and facilitates comparisons of different aspects of a car. DCEs are designed to facilitate CA similarly to real-life examples by presenting 2 options that have the same attributes but with varying values (Figure).

**Figure.**
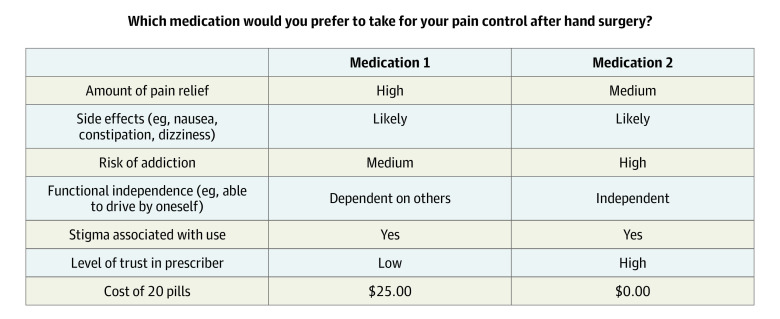
Example of a Discrete-Choice Survey Question

Through a series of DCEs and CA, we sought to calculate patient-perceived utility associated with different attributes of postoperative pain control.^11,12^ When performing conjoint analysis, it is assumed that a product can be described based on a limited number of attributes and that individuals are more likely to choose the option that has greater utility.

### Survey Design

In developing our survey, we adhered to recommendations from the International Society for Pharmacoeconomics and Outcomes Research checklist for conjoint analysis applications in health care.^9^ A preliminary list of attributes was made from a comprehensive literature review and consultation of the hand surgeons at our institution. Subsequently, we conducted qualitative interviews with 30 patients to confirm that all relevant attributes had been identified.^13^ The final list included 7 attributes: amount of pain relief, adverse effects, risk of addiction, functional independence, stigma, level of trust in the prescriber, and cost. Of these attributes, 3 attributes had 3 value levels (ie, high, medium, and low for amount of pain relief, risk of addiction, and cost of 1 prescription [specified as levels of $25, $10, and $0]), and the remaining 4 attributes had 4 value levels (ie, likely vs unlikely for adverse effects, independent vs dependent on others for functional independence, yes vs no for stigma, and high vs low for level of trust in prescriber) (Table 1). Using the Conjoint Analysis Software Tool version 2019 (QCAST; Qualtrics) and logit computation of standard error, we determined that 250 completed surveys would provide adequate statistical power given a 5% margin of error.^14,15,16^

**Table 1.** Attributes and Levels

Attribute	Levels
Amount of pain relief	High
	Medium
	Low
Adverse effects	Likely
	Unlikely
Risk of addiction	High
	Medium
	Low
Functional independence	Independent
	Dependent on others
Stigma	Yes
	No
Level of trust in prescriber	High
	Low
Cost	High
	Medium
	Low

We used QCAST to distribute the online survey. At the start, participants were introduced to the study purpose and estimated time needed for completion of the survey. Informed consent was obtained. Next, participants were asked to imagine that they’d just had elective, outpatient surgical treatment and were presented with a series of DCEs asking them to select their preferred pain medication (Figure). Each question presented a pair of options that were designed algorithmically by QCAST based on participants’ previous choice selections. This minimized the number of questions a participant had to answer while maintaining adequate statistical power to measure interactions between attributes and value levels. We did not specify whether options were opioids.

After completing the DCE portion of the survey, participants were asked to select the type of surgical treatment they had undergone. We also collected participant demographic characteristics, including sex, age, race, ethnicity, employment status, household income, and education level. We collected self-reported race and ethnicity (participants selected their race and ethnicity on the survey) to assess the diversity of the cohort and identify differences in pain control preferences based on patient characteristics.

### Statistical Analysis

We used Qualtrics software to calculate participant part-worth utilities, which are a quantification of patient preferences for each value level of the 7 attributes (Table 1). Qualtrics uses hierarchical bayesian estimation and multinomial logistic regression to generate these values by combining aggregate and individual data. This statistical technique consists of a higher-level model that estimates mean preferences based on a normal distribution. A lower-level model is then used to estimate individuals’ relative utility values by evaluating how their responses vary from the distribution. The outcome of discrete choice experiments includes part-worth utilities for each level of an attribute, with positive values indicating that an attribute level is desirable. Negative values indicate that an attribute level is less desirable and, therefore, less likely to be selected.

After calculating individual participants’ part-worth utilities, we determined mean part-worth utility for each attribute value level across the study population using QCAST. Specifically, attribute importance = ([utility range]/[sum of utility range for all characteristics]) × 100. We determined SDs for attribute importance scores and 95% CIs for part-worth utility values using Microsoft Excel version 2108. We also conducted subgroup analyses for the most common hand conditions in our study population (ie, carpal tunnel syndrome, carpometacarpal arthritis, and trigger finger), as well as sex, age, race, ethnicity, and previous opioid use. We did not conduct subgroup analysis if fewer than 5 responders met subgroup criteria. In addition, we compared demographic characteristics of survey responders and nonresponders. Data were analyzed from May to July 2021.

## Results

We collected 321 completed surveys from 710 invited participants (completion rate, 45.2%) between November 2019 and January 2020. There were 212 (66.0%) women, 108 (33.6%) men, and 1 individual who responded other to sex; the most common age category was 60 to 69 years (102 participants [31.8%]). There were 3 Asian individuals (0.9%), 5 American Indian or Alaska Native individuals, 18 Black individuals (5.6%), 1 Native Hawaiian or other Pacific Islander individual, 2895 White individuals (90.0%), and 5 individuals with other race. There were 8 Hispanic individuals (2.5%). Participants self-selected other if their race or ethnicity was not listed; we did not ask them to specify their race or ethnicity, so groups in other are unknown. Most participants were employed full time (144 individuals [44.9%]) or retired (107 individuals [33.3%]). Responders most frequently underwent surgical treatment for carpal tunnel syndrome (CTS) (107 individuals [33.3%]), carpometacarpal (CMC) arthritis (70 individuals [21.8%]), or trigger finger (62 individuals [19.3%]). Most responders (282 individuals [87.9%]) reported previous opioid use (Table 2).

**Table 2.** Patient Characteristics

Characteristic	Patients, No. (%) (N = 321)
Sex	
Women	212 (66.0)
Men	108 (33.6)
Other	1 (0.3)
Age, y	
18-19	1 (0.3)
20-29	11 (3.4)
30-39	18 (5.6)
40-49	42 (13.1)
50-59	89 (27.7)
60-69	102 (31.8)
70-79	48 (15.0)
≥80	10 (3.1)
Race	
American Indian or Alaska Native	5 (1.6)
Asian	3 (0.9)
Black or African American	18 (5.6)
Native Hawaiian or Other Pacific Islander	1 (0.3)
White	289 (90.0)
Other	5 (1.6)
Ethnicity	
Hispanic	8 (2.5)
Non-Hispanic	313 (97.5)
Employment status	
Employed full time	144 (44.9)
Employed part time	22 (6.9)
Homemaker	9 (2.8)
Student	6 (1.9)
Unemployed	7 (2.2)
On disability	26 (8.1)
Retired	107 (33.3)
Military	0
Household income, $/y	
<30 000	32 (10.0)
30 000-50 000	31 (9.7)
50 001-75 000	36 (11.2)
75 001-100 000	56 (17.4)
>100 000	123 (38.3)
Prefer not to answer	43 (13.4)
Education level	
<8th grade	0
Some high school, no diploma	3 (0.9)
High school graduate or equivalent	19 (5.9)
Some college, no degree	50 (15.6)
Associate’s degree	38 (11.8)
Bachelor’s degree	93 (29.0)
Master’s degree	87 (27.1)
Doctorate degree	31 (9.7)
Hand condition	
Carpal tunnel syndrome	107 (33.3)
Cubital tunnel syndrome	18 (5.6)
Carpometacarpal arthritis	70 (21.8)
Trigger finger	62 (19.3)
Ganglion cyst	34 (10.6)
Benign mass (including mucous cyst)	14 (4.3)
De Quervain tenosynovitis	16 (5.0)
Previous opioid use	
Yes	282 (87.9)
No	39 (12.1)

### Discrete Choice Experiments

Part-worth utility values and attribute importance scores for the entire patient sample are reported in Table 3. Risk of addiction and amount of pain relief had the highest attribute importance scores (SDs), at 26.3% (13.0%) and 25.6% (14.6%), respectively. Part-worth utilities for high, medium, and low risk of addiction were −14.9 (95% CI, −15.8 to −14.0), 3.5 (95% CI, 3.2 to 3.8), and 11.4 (95% CI, 10.6 to 12.2), respectively. The increased value for low risk of addiction suggests that it is more favorable than medium or high risk of addiction. In addition, the negative part-worth utility for low pain relief had a greater absolute value than the positive part-worth utility for high pain relief (high: 10.8 [95% CI, 9.4 to 12.2], medium: 3.9 [95% CI, 3.6 to 4.2], low: −14.7 [95% CI, −16.1 to −13.3]), suggesting that patients were more put off by the prospect of low pain relief than they were compelled by the prospect of high pain relief. The attribute importance scores (SDs) for the likelihood of adverse effects (13.9% [7.2%]), functional independence (11.8% [7.3%]), and level of trust in the clinician (11.4% [5.8%]) were intermediate. Meanwhile, the attribute importance score (SD) of cost was 7.9% (4.4%), and the negative part-worth utility for higher costs had a greater absolute value than the positive part-worth utility for lower costs ($0: 2.9 [95% CI, 2.6 to 3.2]; $10: 2.0 [95% CI, 1.9 to 2.1]; $25: −4.9 [95% CI, −5.2 to −4.6]), suggesting that patients were more deterred by higher costs of opioids than they were incentivized by lower costs. Stigma associated with use had the lowest attribute importance score (SD), at 3.1% (1.3%).

**Table 3.** Overall Attributes

Attribute	Part-worth utility (CI)	Attribute importance, % (SD)
Risk of addiction		26.3 (13.0)
High	−14.9 (−15.8 to −14.0)	
Medium	3.5 (3.2 to 3.8)	
Low	11.4 (10.6 to 12.2)	
Amount of pain relief		25.6 (14.6)
High	10.8 (9.4 to 12.2)	
Medium	3.9 (3.6 to 4.2)	
Low	−14.7 (−16.1 to −13.3)	
Adverse effects		13.9 (7.2)
Likely	−7.0 (−7.4 to −6.6)	
Unlikely	7.0 (6.6 to 7.4)	
Functional independence		11.8 (7.3)
Independent	5.9 (5.5 to 6.3)	
Dependent on others	−5.9 (−6.3 to −5.5)	
Level of trust in clinician		11.4 (5.8)
High	5.7 (5.4 to 6.0)	
Low	−5.7 (−6.0 to −5.4)	
Cost for 1 prescription, $		7.9 (4.4)
0	2.9 (2.6 to 3.2)	
10	2.0 (1.9 to 2.1)	
25	−4.9 (−5.2 to −4.6)	
Stigma associated with use		3.1 (1.3)
Yes	−1.5 (−1.6 to −1.4)	
No	1.5 (1.4 to 1.6)	

When we analyzed the CTS, CMC arthritis, and trigger finger samples separately, risk of addiction and amount of pain relief remained the attributes with the highest attribute importance scores (eTables 2-4 in the Supplement). However, the attribute importance scores (SDs) of pain relief (27.2% [16.3%]) and risk of addiction (26.4% [13.9%]) among participants with CTS were increased compared with those of the overall sample. In addition, patients with CTS and CMC had increased attribute importance scores for pain relief than for risk of addiction, in contrast to patients with trigger finger and the overall patient cohort. Cost and stigma associated with use remained the attributes with the lowest attribute importance scores across the CTS, CMC arthritis, and trigger finger samples. We also analyzed male and female subgroups and observed that the attribute importance score (SD) of risk of addiction was greater than that of pain relief among men (26.4% [12.6%] vs 23.8% [12.6%]) and lower than that of pain relief among women (26.2% [13.3%] vs 26.5% [15.5%]). For the age 20 to 29 years, age 30 to 39 years, and age 80 years and older subgroups, the attribute importance score for risk of addiction was at least 10 points greater than that of pain relief, in contrast to the middle-aged populations. The same trend was observed among the Hispanic subgroup, although the opposite trend was observed among the Asian subgroup. With respect to prior opioid use, the attribute importance score for risk of addiction was increased among patients who were opioid-naive compared with those with previous opioid use (30.6% [14.0%] vs 25.7% [12.8%]). The attribute importance score (SD) of pain control was increased among previous opioid users compared with those who were opioid-naive (26.4% [14.9%] vs 19.6% [10.5%]). All subgroup results are displayed in eTables 2-21 in the Supplement.

We also determined the breakdown of sex, age, race, and ethnicity among 389 individuals who did not respond to the survey and compared these data with the responder cohort demographics. There was an increased proportion of women and White individuals in the responder cohort (212 [66.0%] women vs 240 [61.7%] women and 289 White individuals [90.0%] vs 330 White individuals [84.8%], respectively). Meanwhile, there was a decreased proportion of Black and Hispanic individuals in the responder cohort (18 Black individuals [5.6%] vs 29 Black individuals [7.5%] and 8 Hispanic individuals [2.5%] vs 12 Hispanic individuals [3.1%]). Demographics of nonresponders are displayed in eTable 22 in the Supplement.

## Discussion

Quantifying patient preferences in postoperative pain control may help surgeons gain a robust understanding of patient decision-making processes. Using DCE and CA in this decision analytical model study of patient preferences for postoperative opioid use, we observed that risk of addiction and amount of pain relief were the most important factors in decisions for pain control among patients who underwent elective surgical treatment. Patient level of trust in clinicians, cost, and stigma were less influential attributes. These results suggest that a multimodal approach to pain control that is associated with adequate pain relief and minimized risk of addiction may be preferable for treatment of acute postoperative pain. Identifying procedures for which patients prioritize minimizing risk of addiction over pain relief, such as trigger finger release in this study, may present an opportunity for decreased postoperative opioid prescribing.

Our findings contrast with research on chronic pain, for which patients often rank avoidance of adverse effects as one of the most important factors when devising pain control regimens.^6,7,17^ For example, in a DCE of patients with chronic osteoarthritis, the highest-ranking attribute was symptom control, followed by risk of addiction.^17^ Meanwhile, our study of acute pain control found that the potential for adverse effects was less important than pain relief or risk of addiction. Furthermore, unlike patients with chronic pain, who may use opioids after finding other treatment modalities ineffective, patients with acute postoperative pain are often prescribed opioids despite the availability of effective, yet less addictive medications. For instance, a randomized controlled trial^18^ found that acetaminophen or ibuprofen can provide comparable if not better pain relief than oxycodone after carpal tunnel release. These findings suggest that guidelines are necessary to ensure that patients receive adequate pain control and minimal exposure to addictive substances after surgical treatment.

Determining procedures for which patients prioritize minimizing risk of addiction may aid in decreasing postoperative opioid prescribing, especially given the increasing body of research on multimodal treatments for postoperative pain. In addition to the aforementioned study on CTR, an investigation into patient satisfaction after general surgical procedures,^19^ such as laparoscopic cholecystectomy and thyroidectomy, found that patients were satisfied after using acetaminophen and ibuprofen rather than opioids to control their pain. This opioid-sparing approach may be beneficial considering that up to 10% of patients who are opioid-naive who receive an opioid prescription after surgical treatment may develop physical dependence.^20^ Furthermore, 3 studies^21,22,23^ found that local anesthesia is associated with decreased severity of postoperative pain relative compared with general anesthesia and nerve blocks. Identifying procedures for which local anesthesia or multimodal pain regimens can be used may provide an additional avenue to decrease the need for postoperative opioids.

When patients have a history of opioid misuse or opioid-seeking behavior, clinician expertise is essential to prevent or treat addiction. Meanwhile, in situations in which the risk of addiction is less obvious, patients often receive opioids without consideration of their personal preferences or alternative pain control options. This suggests that patient-centered guidelines for use of multimodal pain control are necessary to ensure that decisions are made in patients’ best interest. Our study findings suggest that patients may desire different attributes of pain control depending on age, race, and ethnicity. For example, Hispanic patients and those aged 20 to 29 years ranked the attribute importance of risk of addiction higher than other subgroups did. It is inappropriate to use demographic characteristics alone to determine pain levels, as various studies have found racism and other forms of discrimination in pain management.^24^ Shared decision-making, in particular, may facilitate patient-physician discussions of pain control. For example, Vilkins et al^25^ used a visual decision aid to ensure that patients received uniform education on posthysterectomy pain management. This intervention was associated with decreased opioid prescribing without a decrease in patient satisfaction. However, similar interventions are needed across surgical treatments to substantially curb unnecessary opioid use.

### Limitations

This study has several limitations, including selection bias, given that responders were recruited from a single institution and underwent hand procedures. However, the findings may be generalized to other outpatient procedures that require medical intervention for pain control. Considering that most responders were White, were not Hispanic, and were college educated, the findings may be less applicable in a more diverse population. Furthermore, given that participants were recruited using email, we likely selected against individuals who are unfamiliar with web-based technology. Given that individuals increasingly seek health information online, it is possible that responders to our survey were more familiar with pain control options than the general population.^26^ However, a comparison of responder and nonresponder cohorts found no substantial differences by key demographic characteristics. Furthermore, research indicates that individuals tend to put less emphasis on future payoffs, in what is known as hyperbolic discounting.^27^ This may have been associated with an overemphasis on value-levels that provided short-term benefits, such as high pain relief over low pain relief or unlikely chance of adverse effects over likely chance of adverse effects. Additionally, we could not determine which medications responders received at discharge, which could have influenced their preferences.

It is also important to consider the limitations inherent to the survey, given that survey fatigue may have prevented responders from fully considering advantages and disadvantages of the choices. Additionally, it was not feasible to test every attribute or value level that may have been relevant. However, we relied on the literature and conducted a qualitative study of perspectives on postoperative opioid use among patients who underwent hand surgical treatment to identify the most important attributes. Additionally, Qualtrics software facilitated complex analysis of survey data.^28^ However, it did not provide measures of variance that may be used to gauge the true value of attribute importance or part-worth utilities across subgroups.

## Conclusions

Although this study provided a quantitative assessment of patient preferences for aspects of pain control, it is not meant to replace evaluations necessary to determine a unique patient’s postoperative needs. Nevertheless, these findings may lay the foundation for surgeon-patient discussions and support the development of decision-aids that incorporate important attributes associated with pain control, such as risk of addiction and adverse effects. Research has found that many patients undergoing surgical treatment depend on their personal beliefs and understanding of opioid addiction when deciding whether to take opioid pills postoperatively; however, these considerations are not consistently taken into account when opioids are prescribed.^13^

Quantifying patient preferences may help surgeons devise pain control regimens that are evidence-based, safe, and effective for individual patients. This study found that patients undergoing elective surgical treatment considered risk of addiction and pain relief the most important aspects of pain control, although their preferences varied based on specific condition or procedure. These findings suggest the importance of using multimodal regimens associated with minimized risk of addiction with sufficient pain relief.

## Notes

Supplement. **eAppendix.** Survey Design **eTable 1.** Current Procedural Terminology (CPT) Codes for Outpatient Procedures Queried **eTable 2.** Carpal Tunnel Attributes **eTable 3.** Carpometacarpal Arthritis Attributes **eTable 4.** Trigger Finger Release Attributes **eTable 5.** Attributes of Men **eTable 6.** Attributes of Women **eTable 7.** Age 20-29 y Attributes **eTable 8.** Age 30-39 y Attributes **eTable 9.** Age 40-49 y Attributes **eTable 10.** Age 50-59 y Attributes **eTable 11.** Age 60-69 y Attributes **eTable 12.** Age 70-79 y Attributes **eTable 13.** Age ≥80 y Attributes **eTable 14.** Attributes of Non-Hispanic Individuals **eTable 15.** Attributes of Hispanic Individuals **eTable 16.** Attributes of White Individuals **eTable 17.** Attributes of Black Individuals **eTable 18.** Attributes of American Indian or Alaska Native Individuals **eTable 19.** Attributes of Asian Individuals **eTable 20.** Previous Opioid Use Attributes **eTable 21.** No Previous Opioid Use Attributes **eTable 22.** Comparison of Responders and Nonresponders
