# Supplementary material for: Evaluation of Factors Relevant to Pain Control Among Patients After Surgical Treatment
Source: JAMA Netw Open. 2021 Dec 28;4(12):e2140869. doi: 10.1001/jamanetworkopen.2021.40869 (PMC8715341; doi:10.1001/jamanetworkopen.2021.40869)
Supplement: Supplement. — eAppendix. Survey Design eTable 1. Current Procedural Terminology (CPT) Codes for Outpatient Procedures Queried eTable 2. Carpal Tunnel Attributes eTable 3. Carpometacarpal Arthritis Attributes eTable 4. Trigger Finger Release Attributes eTable 5. Attributes of Men eTable 6. Attributes of Women eTable 7. Age 20-29 y Attributes eTable 8. Age 30-39 y Attributes eTable 9. Age 40-49 y Attributes eTable 10. Age 50-59 y Attributes eTable 11. Age 60-69 y Attributes eTable 12. Age 70-79 y Attributes eTable 13. Age ≥80 y Attributes eTable 14. Attributes of Non-Hispanic Individuals eTable 15. Attributes of Hispanic Individuals eTable 16. Attributes of White Individuals eTable 17. Attributes of Black Individuals eTable 18. Attributes of American Indian or Alaska Native Individuals eTable 19. Attributes of Asian Individuals eTable 20. Previous Opioid Use Attributes eTable 21. No Previous Opioid Use Attributes eTable 22. Comparison of Responders and Nonresponders [file jamanetwopen-e2140869-s001.pdf]

Supplemental Online Content

Baxter NB, Cho HE, Billig JI, Kotsis SV, Haase SC, Chung KC. Evaluation of factors relevant to pain control among patients after surgical treatment. *JAMA Netw Open*. 2021;4(12):e2140869. doi:10.1001/jamanetworkopen.2021.40869

eAppendix. Survey Design
eTable 1. Current Procedural Terminology (CPT) Codes for Outpatient Procedures Queried
eTable 2. Carpal Tunnel Attributes
eTable 3. Carpometacarpal Arthritis Attributes
eTable 4. Trigger Finger Release Attributes
eTable 5. Attributes of Men
eTable 6. Attributes of Women
eTable 7. Age 20-29 y Attributes
eTable 8. Age 30-39 y Attributes
eTable 9. Age 40-49 y Attributes
eTable 10. Age 50-59 y Attributes
eTable 11. Age 60-69 y Attributes
eTable 12. Age 70-79 y Attributes
eTable 13. Age ≥80 y Attributes
eTable 14. Attributes of Non-Hispanic Individuals
eTable 15. Attributes of Hispanic Individuals
eTable 16. Attributes of White Individuals
eTable 17. Attributes of Black Individuals
eTable 18. Attributes of American Indian or Alaska Native Individuals
eTable 19. Attributes of Asian Individuals
eTable 20. Previous Opioid Use Attributes
eTable 21. No Previous Opioid Use Attributes
eTable 22. Comparison of Responders and Nonresponders

This supplemental material has been provided by the authors to give readers additional information about their work.

eAppendix. Survey Design

We're doing a study to learn more about patients' preferences regarding pain control after surgery. To get information we'd like 250 people to answer this survey. We expect it to take about 10 minutes to complete.

Your input will help us improve the quality of care at Michigan Medicine. Please note that your responses will not impact your clinical care in any way.

If you have any questions, please contact:
Michigan Center for Hand Outcomes and Innovation Research (M-CHOIR)
Phone: 734-763-1829
E-mail: mchoirresearch@umich.edu

---

1. Before we move on, please confirm your age

   I am 18 years of age or older:

   ○ Yes (1)

   ○ No (2)

2. Consent Process
   a. Answering this survey is voluntary. You don't have to answer it if you'd rather not. Choosing not to answer our survey won't affect the medical care you might receive at the University of Michigan Health System.
   b. It's possible that some of the questions may make you feel uncomfortable. If a question makes you uncomfortable, you can just stop the survey.
   c. To keep your information confidential, we will not link your responses to your identity.
   d. Answering our survey won't benefit you directly. We hope what we learn will help other people in the future.
   e. To thank you for taking part in our study, we'll send you a $5 check after you take the survey. The University of Michigan accounting department may need your name, address, and payment amount for tax reporting purposes.
   f. By clicking yes below, you are consenting to participate in this research study.

   ○ Yes (1)

   ○ No (2)

3. For the next 6 questions, please imagine that you just had an outpatient surgery for a minor hand condition (e.g. carpal tunnel, trigger finger). You will be presented with 2 different options for your pain medication. Please review the characteristics of each medication and choose the one you'd prefer to take.

   Refer to Figure 1 for question structure and display of the next 6 questions.

9. What is your sex?

- Male (1)
- Female (2)
- Other (3)

10. What is your age?

- 18-19 (1)
- 20-29 (2)
- 30-39 (3)
- 40-49 (4)
- 50-59 (5)
- 60-69 (6)
- 70-79 (7)
- 80 or older (8)

11. Please specify your ethnicity

- Hispanic (1)
- Non-Hispanic (2)

12. Please specify your race (pick ONE that best describes you)

© 2021 Baxter NB et al. *JAMA Network Open.*

- White (1)
- Black or African American (2)
- American Indian or Alaska Native (3)
- Asian (4)
- Native Hawaiian or Pacific Islander (5)
- Other (6)

13. What is your current employment status? Please pick ONE that best describes your status.

- Employed full-time (1)
- Employed part-time (2)
- Student (3)
- Military (4)
- Homemaker (5)
- Unemployed (6)
- On disability (7)
- Retired (8)

© 2021 Baxter NB et al. *JAMA Network Open.*

14. What is your total annual household income?

- ◯ Less than $30,000 (1)
- ◯ $30,000 - $50,000 (2)
- ◯ $50,001 - $75,000 (3)
- ◯ $75,001 - $100,000 (4)
- ◯ More than $100,000 (5)
- ◯ Prefer not to answer (6)

15. What is the highest level of school you completed?

- ◯ Less than 8th grade (1)
- ◯ Some high school, no diploma (2)
- ◯ High school graduate or equivalent (3)
- ◯ Some college, no degree (4)
- ◯ Associate degree (5)
- ◯ Bachelor's degree (6)
- ◯ Master's degree (7)
- ◯ Doctorate degree (8)

© 2021 Baxter NB et al. *JAMA Network Open.*

16. What kind of hand condition do you have? Please pick ONE diagnosis that has/had the most impact in your daily living.

- ○ Carpal tunnel syndrome (1)
- ○ Cubital tunnel syndrome (also called: ulnar neuropathy at the elbow) (2)
- ○ Thumb arthritis (also called: basilar thumb (CMC) arthritis) (3)
- ○ Trigger finger (4)
- ○ Ganglion cyst (5)
- ○ Benign mass (including mucous cyst) (6)
- ○ De Quervain's tenosynovitis (also called: radial styloid tenosynovitis) (7)

17. Have you ever taken opioid (narcotic) pain medications (e.g. Vicodin, Percocet, Norco, Oxycontin, Tramadol) before? It doesn't have to be related to your hand condition (e.g. dental procedures).

- ○ Yes (1)
- ○ No (2)

Thank you very much for your input! This is the end of the survey.

To receive your payment, please click the NEXT button.

eTable 1. Current Procedural Terminology (CPT) Codes for Outpatient Procedures Queried

Procedure category	CPT code	Description
Carpal Tunnel Release	29848	Carpal tunnel release, endoscopic
	64721	Neuroplasty and/or transposition; median nerve at carpal tunnel
Cubital Tunnel Release	64718	Neuroplasty and/or transposition; ulnar nerve at elbow
CMC Arthroplasty	20600	Arthrocentesis, aspiration and/or injection; small joint, bursa or ganglion cyst eg, fingers, toes
	20924	Tendon graft, from a distance eg, palmaris, toe extensor, plantaris)
	25210	Carpectomy, one bone
	25310	Tendon transplantation or transfer, flexor or extensor, forearm and/or wrist, single; each tendon
	25441	Arthroplasty with prosthetic replacement, distal radius
	25445	Arthroplasty with prosthetic replacement, trapezium
	25447	Interposition arthroplasty, intercarpal or carpometacarpal joints
	25820	Intercarpal fusion; without bone graft
	25825	Intercarpal fusion; with autograft
	26480	Tendon transfer or transplant, carpometacarpal area or dorsum of hand, single; without free graft, each
	26483	tendon transfer or transplant, carpometacarpal area or dorsum of hand, single; with free graft, each
	26820	Fusion in opposition, thumb, with autogenous graft
	26841	Arthrodesis, carpometacarpal joint, thumb, with or without internal fixation
	26842	Arthrodesis, carpometacarpal joint, thumb, with or without internal fixation; with autograft (includes obtaining graft)
Trigger Finger Release	26055	Tendon sheath incision, e.g., for trigger finger
	26145	Synovectomy tendon sheath, radical tenosynovectomy, flexor, palm or finger, single, each digit
	26160	Excision of lesion of tendon sheath or capsule eg, cyst, mucous cyst, or ganglion), hand or finger
Ganglion Excision	20612	aspiration or injection for ganglion cyst
	25110	Excision, lesion of tendon sheath, forearm and/or wrist
	25111	Excision of ganglion, wrist, primary
	25112	Excision of ganglion, wrist, recurrent
	26160	excision of lesion of tendon sheath or capsule, cyst, mucous cyst, or ganglion, hand or finger
Benign/Mucous Cyst Excision	26110	arthrotomy for synovial biopsy, interphalangeal joint, each
	26160	excision of lesion of tendon sheath or capsule, cyst, mucous cyst, or ganglion, hand or finger
	11400	Excision, benign lesion, except skin tag (unless listed elsewhere), trunk, arms or legs; lesion diameter 0.5cm or less
	11401	Excision, benign lesion, except skin tag (unless listed elsewhere), trunk, arms or legs; lesion diameter 0.6 to 1.0cm
	11402	Excision, benign lesion, except skin tag (unless listed elsewhere), trunk, arms or legs; lesion diameter 1.1 to 2.0cm

	11403	Excision, benign lesion, except skin tag (unless listed elsewhere), trunk, arms, or legs; lesion diameter 2.1 to 3.0cm
	11404	Excision, benign lesion, except skin tag (unless listed elsewhere), trunk, arms or legs; lesion diameter 3.1 to 4.0cm
	11406	Excision, benign lesion, except skin tag (unless listed elsewhere), trunk, arms or legs; lesion diameter over 4.0cm
	11420	Excision, benign lesion, except skin tag (unless listed elsewhere), scalp, neck, hands, feet, genitalia; lesion diameter 0.5cm or less
	11421	Excision, benign lesion, except skin tag (unless listed elsewhere), scalp, neck, hands, feet, genitalia; lesion diameter 0.6 to 1.0 cm
	11422	Excision, benign lesion, except skin tag (unless listed elsewhere), scalp, neck, hands, feet, genitalia; lesion diameter 1.1 to 2.0cm
	11423	Excision, benign lesion, except skin tag (unless listed elsewhere), scalp, neck, hands, feet, genitalia; lesion diameter 2.1 to 3.0cm
	11424	Excision, benign lesion, except skin tag (unless listed elsewhere), scalp, neck, hands, feet, genitalia; lesion diameter 3.1 to 4.0cm
	11426	Excision, benign lesion, except skin tag (unless listed elsewhere), scalp, neck, hands, feet, genitalia; lesion diameter over 4.0cm
	24071	Excision, subcutaneous soft tissue tumor; upper arm or elbow, 3cm or greater
	24073	Excision, tumor, upper arm or elbow area; deep, subfascial or intramuscular, 5cm or greater
	24075	Excision subcutaneous soft tissue tumor; upper arm or elbow , less than 3cm
	24076	Excision, tumor, upper arm or elbow area; deep, subfascial or intramuscular, less than 5cm
	24105	Excision, olecranon bursa
	24110	Excision or curettage of bone cyst or benign tumor; humerus
	24115	Excision or curettage of bone cyst or benign tumor; humerus; with autograft includes graft harvest
	24116	Excision or curettage of bone cyst or benign tumor; humerus; with allograft
	24120	Excision or curettage of bone cyst or benign tumor; radial head or neck or olecranon process
	25071	Excision, tumor, forearm and/or wrist area; subcutaneous, 3cm or greater
	25073	Excision, tumor, forearm and/or wrist area; deep, subfascial or intramuscular, 3cm or greater
	25075	Excision, tumor, forearm and/or wrist area; subcutaneous, less than 3cm
	25076	Excision, tumor, forearm and/or wrist area; deep, subfascial or intramuscular, less than 3cm
	25109	Excision of tendon, forearm and/or wrist, flexor or extensor, each

© 2021 Baxter NB et al. *JAMA Network Open.*

	25110	Excision, lesion of tendon sheath, forearm and/or wrist
	25118	Synovectomy, extensor tendon sheath, wrist, single compartment
	25119	Synovectomy, extensor tendon sheath, wrist, single compartment; with resection of distal ulna
	25120	Excision or curettage of bone cyst or benign tumor of radius or ulna (excluding head or neck of radius and
	25125	Excision or curettage of bone cyst or benign tumor of radius or ulna (excluding head or neck of radius and olecranon process); with auto graft (includes obtaining graft)
	25126	Excision or curettage of bone cyst or benign tumor of radius or ulna (excluding head or neck of radius and olecranon process); with allograft
	25130	Excision or curettage of bone cyst or benign tumor of carpal bones
	26111	Excision, tumor or vascular malformation, hand or finger; subcutaneous, 1.5cm or greater
	26113	Excision, tumor or vascular malformation, hand or finger; deep, subfascial, intramuscular, 1.5cm or greater
	26115	Excision, tumor or vascular malformation, hand or finger; subcutaneous, less than 1.5cm
	26116	Excision, tumor or vascular malformation, hand or finger; deep, subfascial, intramuscular, less than 1.5cm
	26160	Excision of lesion of tendon sheath or capsule e.g., cyst, mucous cyst, or ganglion), hand or finger
	26200	Excision or curettage of bone cyst or benign tumor of metacarpal
	26210	Excision or curettage of bone cyst or benign tumor of proximal, middle or distal phalanx of finger
De Quervain Release	25000	Tendon sheath incision, at radial styloid, e.g., for De Quervain disease

© 2021 Baxter NB et al. *JAMA Network Open.*

eTable 2. Carpal Tunnel Attributes

Attribute	Part-worth utility (CI)	Attribute importance, % (SD)
**Risk of addiction**		26.4 (13.9)
High	−14.8 (−15.8 to −13.8)	
Medium	3.3 (3.0 to 3.6)	
Low	11.6 (10.7 to 12.5)	
**Amount of pain relief**		27.2 (16.3)
High	11.7 (10.1 to 13.3)	
Medium	3.9 (3.6 to 4.2)	
Low	−15.6 (-17.2 to -14.0)	
**Side effects**		13.3 (6.4)
Likely	−6.6 (-7.0 to −6.2)	
Unlikely	6.6 (6.2 to 7.0)	
**Functional independence**		11.1 (7.4)
Independent	5.6 (5.2 to 6.0)	
Dependent on others	−5.6 (−6.0 to −5.2)	
**Level of trust in provider**		11.2 (5.3)
High	5.6 (5.3 to 5.9)	
Low	−5.6 (−5.9 to −5.3)	
**Cost**		7.7 (3.7)
$0	2.9 (2.6 to 3.2)	
$10	1.9 (1.8 to 2.0)	
$25	−4.8 (−5.1 to −4.5)	
**Stigma assoc. with use**		3.0 (1.4)
Yes	−1.5 (−1.6 to −1.4)	
No	1.5 (1.4 to 1.6)	

© 2021 Baxter NB et al. *JAMA Network Open.*

eTable 3. Carpometacarpal Arthritis Attributes

Attribute	Part-worth utility (CI)	Attribute importance, % (SD)
**Risk of addiction**		25.5 (13.6)
High	−14.4 (−15.3 to −13.5)	
Medium	3.4 (3.2 to 3.6)	
Low	11.1 (10.2 to 12.0)	
**Amount of pain relief**		26.4 (14.5)
High	11.5 (10.3 to 12.7)	
Medium	3.4 (3.1 to 3.7)	
Low	−14.9 (−16.2 to −13.6)	
**Side effects**		13.4 (6.8)
Likely	−6.7 (−7.1 to −6.3)	
Unlikely	6.7 (6.3 to 7.1)	
**Functional independence**		11.9 (6.9)
Independent	6.0 (5.7 to 6.3)	
Dependent on others	−6.0 (−6.3 to −5.7)	
**Level of trust in provider**		11.6 (6.0)
High	5.8 (5.5 to 6.1)	
Low	−5.8 (−6.1 to −5.5)	
**Cost**		8.1 (5.2)
$0	3.0 (2.6 to 3.4)	
$10	2.0 (1.9 to 2.1)	
$25	−5.0 (−5.3 to −4.7)	
**Stigma assoc. with use**		3.1 (1.4)
Yes	−1.5 (−1.6 to −1.4)	
No	1.5 (1.4 to 1.6)	

© 2021 Baxter NB et al. *JAMA Network Open.*

eTable 4. Trigger Finger Release Attributes

Attribute	Part-worth utility (CI)	Attribute importance, % (SD)
**Risk of addiction**		26.8 (13.1)
High	−15.3 (−16.2 to −14.4)	
Medium	3.7 (3.4 to 4.0)	
Low	11.5 (10.7 to 12.3)	
**Amount of pain relief**		23.1 (13.2)
High	9.2 (7.6 to 10.8)	
Medium	4.7 (4.3 to 5.1)	
Low	−13.9 (−15.6 to −12.2)	
**Side effects**		15.1 (8.2)
Likely	−7.6 (−8.1 to −7.1)	
Unlikely	7.6 (7.1 to 8.1)	
**Functional independence**		11.6 (6.9)
Independent	5.8 (5.5 to 6.1)	
Dependent on others	−5.8 (−6.1 to −5.5)	
**Level of trust in provider**		11.1 (5.7)
High	5.6 (5.3 to 5.9)	
Low	−5.6 (−5.9 to −5.3)	
**Cost**		9.0 (5.6)
$0	3.5 (3.2 to 3.8)	
$10	2.1 (2.0 to 2.2)	
$25	−5.5 (−5.8 to −5.2)	
**Stigma assoc. with use**		3.3 (1.4)
Yes	−1.6 (−1.7 to −1.5)	
No	1.6 (1.5 to 1.7)	

© 2021 Baxter NB et al. *JAMA Network Open.*

eTable 5. Attributes of Men

Attribute	Part-worth utility (CI)	Attribute importance, % (SD)
**Risk of addiction**		26.4 (12.6)
High	−15.0 (−15.9 to −14.1)	
Medium	3.6 (3.3 to 3.9)	
Low	11.4 (10.6 to 12.2)	
**Amount of pain relief**		23.8 (12.6)
High	9.6 (8.0 to 11.2)	
Medium	4.6 (4.2 to 5.0)	
Low	−14.2 (−15.9 to −12.5)	
**Side effects**		14.5 (7.1)
Likely	−7.2 (−7.6 to −6.8)	
Unlikely	7.2 (6.8 to 7.6)	
**Functional independence**		12.4 (7.3)
Independent	6.2 (5.8 to 6.6)	
Dependent on others	−6.2 (−6.6 to −5.8)	
**Level of trust in provider**		11.6 (5.5)
High	5.8 (5.5 to 6.1)	
Low	−5.8 (−6.1 to −5.5)	
**Cost**		8.0 (4.5)
$0	2.9 (2.5 to 3.3)	
$10	2.1 (2.0 to 2.2)	
$25	−5.0 (−5.3 to −4.7)	
**Stigma assoc. with use**		3.3 (1.4)
Yes	−1.7 (−1.8 to −1.6)	
No	1.7 (1.6 to 1.8)	

© 2021 Baxter NB et al. *JAMA Network Open.*

eTable 6. Attributes of Women

Attribute	Part-worth utility (CI)	Attribute importance, % (SD)
**Risk of addiction**		26.2 (13.3)
High	−14.8 (−15.8 to −13.8)	
Medium	3.5 (3.2 to 3.8)	
Low	11.4 (10.5 to 12.3)	
**Amount of pain relief**		26.5 (15.5)
High	11.4 (10.1 to 12.7)	
Medium	3.6 (3.3 to 3.9)	
Low	−15.0 (−16.3 to −13.7)	
**Side effects**		13.7 (7.3)
Likely	−6.8 (−7.2 to −6.4)	
Unlikely	6.8 (6.4 to 7.2)	
**Functional independence**		11.5 (7.3)
Independent	5.8 (5.4 to 6.2)	
Dependent on others	−5.8 (−6.2 to −5.4)	
**Level of trust in provider**		11.3 (5.9)
High	5.7 (5.4 to 6.0)	
Low	−5.7 (−6.0 to −5.4)	
**Cost**		7.8 (4.4)
$0	2.9 (2.6 to 3.2)	
$10	1.9 (1.8 to 2.0)	
$25	−4.9 (−5.2 to −4.6)	
**Stigma assoc. with use**		3.1 (1.3)
Yes	−1.5 (−1.6 to −1.4)	
No	1.5 (1.4 to 1.6)	

© 2021 Baxter NB et al. *JAMA Network Open.*

eTable 7. Age 20-29 y Attributes

Attribute	Part-worth utility (CI)	Attribute importance, % (SD)
**Risk of addiction**		35.8 (14.0)
High	−19.8 (−20.6 to −19.0)	
Medium	3.9 (3.6 to 4.2)	
Low	16.0 (15.3 to 16.7)	
**Amount of pain relief**		17.9 (11.4)
High	7.6 (6.4 to 8.8)	
Medium	2.8 (2.2 to 3.6)	
Low	−10.3 (−11.6 to −9.0)	
**Side effects**		13.5 (6.8)
Likely	−6.8 (−7.2 to −6.4)	
Unlikely	6.8 (6.4 to 7.2)	
**Functional independence**		10.9 (9.8)
Independent	5.4 (4.9 to 5.9)	
Dependent on others	−5.4 (−5.9 to −4.9)	
**Level of trust in provider**		11.8 (4.9)
High	5.9 (5.3 to 6.5)	
Low	−5.9 (−6.5 to −5.3)	
**Cost**		6.6 (1.9)
$0	2.3 (2.1 to 2.5)	
$10	2.1 (2.0 to 2.2)	
$25	−4.3 (−4.5 to −4.1)	
**Stigma assoc. with use**		3.5 (1.0)
Yes	−1.7 (−1.7 to −1.7)	
No	1.7 (1.7 to 1.7)	

© 2021 Baxter NB et al. *JAMA Network Open.*

eTable 8. Age 30-39 y Attributes

Attribute	Part-worth utility (CI)	Attribute importance, % (SD)
**Risk of addiction**		30.6 (11.7)
High	−17.0 (−17.9 to −16.1)	
Medium	3.3 (3.0 to 3.6)	
Low	13.7 (12.9 to 14.5)	
**Amount of pain relief**		20.5 (14.2)
High	8.7 (7.2 to 10.2)	
Medium	3.1 (2.7 to 3.5)	
Low	−11.8 (−13.3 to −10.3)	
**Side effects**		13.8 (6.0)
Likely	−6.9 (−7.2 to −6.6)	
Unlikely	6.9 (6.6 to 7.2)	
**Functional independence**		12.9 (6.5)
Independent	6.5 (6.2 to 6.8)	
Dependent on others	−6.5 (−6.8 to −6.2)	
**Level of trust in provider**		11.1 (5.9)
High	5.6 (5.3 to 5.9)	
Low	−5.6 (−5.9 to −5.3)	
**Cost**		7.8 (3.8)
$0	3.0 (2.7 to 3.3)	
$10	1.8 (1.7 to 1.9)	
$25	−4.8 (−5.1 to −4.5)	
**Stigma assoc. with use**		3.2 (1.3)
Yes	−1.6 (−1.7 to −1.5)	
No	1.6 (1.5 to 1.7)	

© 2021 Baxter NB et al. *JAMA Network Open.*

eTable 9. Age 40-49 y Attributes

Attribute	Part-worth utility (CI)	Attribute importance, % (SD)
**Risk of addiction**		23.7 (15.1)
High	−13.6 (−14.2 to −13.0)	
Medium	3.4 (3.3 to 3.5)	
Low	10.2 (9.6 to 10.8)	
**Amount of pain relief**		28.5 (15.2)
High	12.2 (10.7 to 13.7)	
Medium	4.2 (3.9 to 4.5)	
Low	−16.4 (−17.9 to −14.9)	
**Side effects**		13.1 (7.4)
Likely	−6.6 (−6.9 to −6.3)	
Unlikely	6.6 (6.3 to 6.9)	
**Functional independence**		11.2 (6.3)
Independent	5.6 (5.3 to 5.9)	
Dependent on others	−5.6 (−5.9 to −5.3)	
**Level of trust in provider**		12.4 (5.5)
High	6.2 (5.9 to 6.5)	
Low	−6.2 (−6.5 to −5.9)	
**Cost**		8.3 (6.9)
$0	3.2 (2.8 to 3.6)	
$10	1.8 (1.7 to 1.9)	
$25	−5.0 (−5.3 to −4.7)	
**Stigma assoc. with use**		2.8 (1.1)
Yes	−1.4 (−1.5 to −1.3)	
No	1.4 (1.3 to 1.5)	

© 2021 Baxter NB et al. *JAMA Network Open.*

eTable 10. Age 50-59 y Attributes

Attribute	Part-worth utility (CI)	Attribute importance, % (SD)
**Risk of addiction**		25.5 (13.4)
High	−14.5 (−15.7 to −13.3)	
Medium	3.5 (3.2 to 3.8)	
Low	11.0 (9.9 to 12.1)	
**Amount of pain relief**		25.3 (16.3)
High	10.8 (9.6 to 12.0)	
Medium	3.7 (3.4 to 4.0)	
Low	−14.5 (−15.8 to −13.2)	
**Side effects**		14.3 (6.6)
Likely	−7.1 (−7.5 to −6.7)	
Unlikely	7.1 (6.7 to 7.5)	
**Functional independence**		12.8 (8.2)
Independent	6.4 (5.9 to 6.9)	
Dependent on others	−6.4 (−6.9 to −5.9)	
**Level of trust in provider**		11.1 (5.7)
High	5.6 (5.3 to 5.9)	
Low	−5.6 (−5.9 to −5.3)	
**Cost**		7.9 (4.4)
$0	2.9 (2.6 to 3.2)	
$10	2.2 (2.1 to 2.3)	
$25	−5.0 (−5.3 to −4.7)	
**Stigma assoc. with use**		3.1 (1.4)
Yes	−1.6 (−1.7 to −1.5)	
No	1.6 (1.5 to 1.7)	

© 2021 Baxter NB et al. *JAMA Network Open.*

eTable 11. Age 60-69 y Attributes

Attribute	Part-worth utility (CI)	Attribute importance, % (SD)
**Risk of addiction**		25.5 (12.4)
High	−14.5 (−15.4 to −13.6)	
Medium	3.5 (3.2 to 3.8)	
Low	11.0 (10.2 to 11.8)	
**Amount of pain relief**		25.3 (12.6)
High	10.8 (9.6 to 12.0)	
Medium	3.7 (3.4 to 4.0)	
Low	−14.5 (−15.8 to −13.2)	
**Side effects**		14.3 (7.9)
Likely	−7.1 (−7.5 to −6.7)	
Unlikely	7.1 (6.7 to 7.5)	
**Functional independence**		12.8 (7.2)
Independent	6.4 (6.0 to 6.8)	
Dependent on others	−6.4 (−6.8 to −6.0)	
**Level of trust in provider**		11.1 (5.4)
High	5.6 (5.3 to 5.9)	
Low	−5.6 (−5.9 to −5.3)	
**Cost**		7.9 (5.1)
$0	2.9 (2.5 to 3.3)	
$10	2.2 (2.1 to 2.3)	
$25	−5.0 (−5.3 to −4.7)	
**Stigma assoc. with use**		3.1 (1.3)
Yes	−1.6 (−1.7 to −1.5)	
No	1.6 (1.5 to 1.7)	

© 2021 Baxter NB et al. *JAMA Network Open.*

eTable 12. Age 70-79 y Attributes

Attribute	Part-worth utility (CI)	Attribute importance, % (SD)
**Risk of addiction**		26.8 (13.4)
High	−15.2 (−16.1 to −14.3)	
Medium	3.6 (3.3 to 3.9)	
Low	11.6 (10.8 to 12.4)	
**Amount of pain relief**		25.9 (14.7)
High	10.8 (9.2 to 12.4)	
Medium	4.3 (3.9 to 4.7)	
Low	−15.1 (−16.7 to −13.5)	
**Side effects**		14.1 (7.1)
Likely	−7.1 (−7.5 to −6.7)	
Unlikely	7.1 (6.7 to 7.5)	
**Functional independence**		10.5 (6.3)
Independent	5.2 (4.8 to 5.6)	
Dependent on others	−5.2 (−5.6 to −4.8)	
**Level of trust in provider**		11.5 (7.1)
High	5.7 (5.4 to 6.0)	
Low	−5.7 (−6.0 to −5.4)	
**Cost**		8.0 (4.3)
$0	3.0 (2.7 to 3.3)	
$10	1.9 (1.8 to 2.0)	
$25	−4.9 (−5.2 to −4.6)	
**Stigma assoc. with use**		3.2 (1.3)
Yes	−1.6 (−1.7 to −1.5)	
No	1.6 (1.5 to 1.7)	

© 2021 Baxter NB et al. *JAMA Network Open.*

eTable 13. Age ≥80 y Attributes

Attribute	Part-worth utility (CI)	Attribute importance, % (SD)
**Risk of addiction**		30.6 (14.6)
High	−16.7 (−17.6 to −15.8)	
Medium	2.7 (2.4 to 3.0)	
Low	14.0 (13.2 to 14.8)	
**Amount of pain relief**		16.5 (12.2)
High	5.9 (2.7 to 9.1)	
Medium	4.7 (3.8 to 5.6)	
Low	−10.6 (−13.3 to −7.9)	
**Side effects**		14.2 (7.2)
Likely	−7.1 (−7.5 to −6.7)	
Unlikely	7.1 (6.7 to 7.5)	
**Functional independence**		14.5 (7.3)
Independent	7.2 (6.8 to 7.6)	
Dependent on others	−7.2 (−7.6 to −6.8)	
**Level of trust in provider**		10.5 (5.6)
High	5.3 (4.9 to 5.7)	
Low	−5.3 (−5.7 to −4.9)	
**Cost**		9.8 (4.0)
$0	3.6 (2.9 to 4.3)	
$10	2.7 (2.6 to 2.8)	
$25	−6.3 (−6.9 to −5.7)	
**Stigma assoc. with use**		3.8 (1.4)
Yes	−1.9 (−2.0 to −1.8)	
No	1.9 (1.8 to 2.0)	

© 2021 Baxter NB et al. *JAMA Network Open.*

eTable 14. Attributes of Non-Hispanic Individuals

Attribute	Part-worth utility (CI)	Attribute importance, % (SD)
**Risk of addiction**		26.0 (12.9)
High	−14.7 (−15.6 to −13.8)	
Medium	3.5 (3.2 to 3.8)	
Low	11.2 (10.4 to 12.0)	
**Amount of pain relief**		25.9 (14.7)
High	11.0 (9.6 to 12.4)	
Medium	3.9 (3.6 to 4.2)	
Low	−14.9 (−16.3 to −13.5)	
**Side effects**		13.9 (7.2)
Likely	−6.9 (−7.3 to −6.5)	
Unlikely	6.9 (6.5 to 7.3)	
**Functional independence**		11.9 (7.4)
Independent	5.9 (5.5 to 6.3)	
Dependent on others	−5.9 (−6.3 to −5.5)	
**Level of trust in provider**		11.5 (5.8)
High	5.7 (5.4 to 6.0)	
Low	−5.7 (−6.0 to −5.4)	
**Cost**		7.9 (4.5)
$0	2.9 (2.6 to 3.2)	
$10	2.0 (1.9 to 2.1)	
$25	−4.9 (−5.2 to −4.6)	
**Stigma assoc. with use**		3.1 (1.4)
Yes	−1.5 (−1.6 to −1.4)	
No	1.5 (1.4 to 1.6)	

© 2021 Baxter NB et al. *JAMA Network Open.*

eTable 15. Attributes of Hispanic Individuals

Attribute	Part-worth utility (CI)	Attribute importance, % (SD)
**Risk of addiction**		39.0 (13.3)
High	−21.6 (−22.4 to −20.8)	
Medium	4.2 (3.9 to 4.5)	
Low	17.4 (16.5 to 18.3)	
**Amount of pain relief**		14.2 (5.0)
High	4.1 (2.4 to 5.8)	
Medium	5.1 (4.2 to 6.0)	
Low	−9.1 (−11.1 to −7.1)	
**Side effects**		16.9 (9.0)
Likely	−8.5 (−9.0 to −8.0)	
Unlikely	8.5 (8.0 to 9.0)	
**Functional independence**		9.1 (3.9)
Independent	4.6 (4.4 to 4.8)	
Dependent on others	−4.6 (−4.8 to −4.6)	
**Level of trust in provider**		9.1 (4.9)
High	4.6 (4.3 to 4.9)	
Low	−4.6 (−4.9 to −4.3)	
**Cost**		8.1 (2.5)
$0	3.1 (2.9 to 3.3)	
$10	1.9 (1.8 to 2.0)	
$25	−5.0 (−5.2 to −4.8)	
**Stigma assoc. with use**		3.4 (0.9)
Yes	−1.7 (−1.8 to −1.6)	
No	1.7 (1.6 to 1.8)	

© 2021 Baxter NB et al. *JAMA Network Open.*

eTable 16. Attributes of White Individuals

Attribute	Part-worth utility (CI)	Attribute importance, % (SD)
**Risk of addiction**		26.5 (13.2)
High	−15.0 (−15.9 to −14.0)	
Medium	3.5 (3.2 to 3.8)	
Low	11.5 (10.7 to 12.3)	
**Amount of pain relief**		25.5 (14.6)
High	10.8 (9.4 to 12.2)	
Medium	3.9 (3.6 to 4.2)	
Low	−14.7 (−16.1 to −13.3)	
**Side effects**		14.1 (7.1)
Likely	−7.0 (−7.4 to −6.6)	
Unlikely	7.0 (6.6 to 7.4)	
**Functional independence**		11.8 (7.2)
Independent	5.9 (5.5 to 6.3)	
Dependent on others	−5.9 (−6.3 to −5.5)	
**Level of trust in provider**		11.3 (5.9)
High	5.6 (5.3 to 5.9)	
Low	−5.6 (−5.9 to −5.3)	
**Cost**		7.7 (4.3)
$0	2.9 (2.6 to 3.2)	
$10	2.0 (1.9 to 2.1)	
$25	−4.9 (−5.2 to −4.6)	
**Stigma assoc. with use**		3.1 (1.3)
Yes	−1.6 (−1.7 to −1.5)	
No	1.6 (1.5 to 1.7)	

© 2021 Baxter NB et al. *JAMA Network Open.*

eTable 17. Attributes of Black Individuals

Attribute	Part-worth utility (CI)	Attribute importance, % (SD)
**Risk of addiction**		24.7 (11.6)
High	−13.9 (−15.2 to −12.6)	
Medium	3.5 (3.0 to 4.0)	
Low	10.4 (9.3 to 11.5)	
**Amount of pain relief**		24.3 (13.2)
High	10.8 (9.9 to 11.7)	
Medium	3.1 (2.8 to 3.4)	
Low	−13.9 (−14.8 to −13.0)	
**Side effects**		14.0 (8.5)
Likely	−7.0 (−7.5 to −6.5)	
Unlikely	7.0 (6.5 to 7.5)	
**Functional independence**		12.0 (8.7)
Independent	6.0 (5.5 to 6.5)	
Dependent on others	−6.0 (−6.5 to −5.5)	
**Level of trust in provider**		12.3 (6.6)
High	6.2 (5.8 to 6.6)	
Low	−6.2 (−6.6 to −5.8)	
**Cost**		9.9 (6.6)
$0	4.0 (3.5 to 4.5)	
$10	1.9 (1.8 to 2.0)	
$25	−5.9 (−6.3 to −5.5)	
**Stigma assoc. with use**		2.7 (1.5)
Yes	−1.3 (−1.4 to −1.2)	
No	1.3 (1.2 to 1.4)	

eTable 18. Attributes of American Indian or Alaska Native Individuals

Attribute	Part-worth utility	Attribute importance, %
**Risk of addiction**		27.5 (11.5)
High	−14.7 (−15.4 to −14.0)	
Medium	1.9 (1.6 to 2.2)	
Low	12.8 (12.1 to 13.5)	
**Amount of pain relief**		23.8 (10.4)
High	10.4 (8.3 to 12.5)	
Medium	3.0 (2.7 to 3.3)	
Low	−13.4 (−15.7 to −11.1)	
**Side effects**		9.8 (7.2)
Likely	−4.9 (−5.3 to −4.5)	
Unlikely	4.9 (4.5 to 5.3)	
**Functional independence**		13.9 (9.3)
Independent	7.0 (6.5 to 7.5)	
Dependent on others	−7.0 (−7.5 to −6.5)	
**Level of trust in provider**		13.8 (4.3)
High	6.9 (6.7 to 7.1)	
Low	−6.9 (−7.1 to −6.7)	
**Cost**		7.8 (3.0)
$0	3.3 (3.2 to 3.4)	
$10	1.3 (1.2 to 1.4)	
$25	−4.6 (−4.7 to −4.5)	
**Stigma assoc. with use**		3.4 (0.8)
Yes	−1.7 (−1.8 to −1.6)	
No	1.7 (1.6 to 1.8)	

eTable 19. Attributes of Asian Individuals

Attribute	Part-worth utility	Attribute importance, %
**Risk of addiction**		16.3 (13.8)
High	−9.6 (−11.0 to −8.2)	
Medium	3.0 (2.6 to 3.4)	
Low	6.6 (5.6 to 7.6)	
**Amount of pain relief**		38.3 (24.0)
High	17.6 (16.2 to 19.0)	
Medium	3.1 (3.0 to 3.2)	
Low	−20.7 (−22.1 to −19.3)	
**Side effects**		13.4 (10.9)
Likely	−6.7 (−7.3 to −6.1)	
Unlikely	6.7 (6.1 to 7.3)	
**Functional independence**		11.9 (1.9)
Independent	5.9 (5.8 to 6.0)	
Dependent on others	−5.9 (−6.0 to −5.8)	
**Level of trust in provider**		11.0 (3.7)
High	5.5 (5.3 to 5.7)	
Low	−5.5 (−5.7 to −5.3)	
**Cost**		6.6 (1.2)
$0	2.4 (2.3 to 2.5)	
$10	1.8 (1.7 to 1.9)	
$25	−4.2 (−4.3 to −4.1)	
**Stigma assoc. with use**		2.6 (1.5)
Yes	−1.3 (−1.4 to −1.2)	
No	1.3 (1.2 to 1.4)	

© 2021 Baxter NB et al. *JAMA Network Open.*

eTable 20. Previous Opioid Use Attributes

Attribute	Part-worth utility (CI)	Attribute importance, % (SD)
**Risk of addiction**		25.7 (12.8)
High	−14.6 (−15.5 to −13.7)	
Medium	3.5 (3.2 to 3.8)	
Low	11.1 (10.3 to 11.9)	
**Amount of pain relief**		26.4 (14.9)
High	11.3 (10.0 to 12.6)	
Medium	3.8 (3.5 to 4.1)	
Low	−15.1 (−16.5 to −13.7)	
**Side effects**		14.0 (7.2)
Likely	−7.0 (−7.4 to −6.6)	
Unlikely	7.0 (6.6 to 7.4)	
**Functional independence**		11.8 (7.2)
Independent	5.9 (5.5 to 6.3)	
Dependent on others	−5.9 (−6.3 to −5.5)	
**Level of trust in provider**		11.3 (5.9)
High	5.7 (5.4 to 6.0)	
Low	−5.7 (−6.0 to −5.4)	
**Cost**		7.8 (4.5)
$0	2.9 (2.6 to 3.2)	
$10	2.0 (1.9 to 2.1)	
$25	−4.9 (−5.2 to −4.6)	
**Stigma assoc. with use**		3.1 (1.4)
Yes	−1.5 (−1.6 to −1.4)	
No	1.5 (1.4 to 1.6)	

© 2021 Baxter NB et al. *JAMA Network Open.*

eTable 21. No Previous Opioid Use Attributes

Attribute	Part-worth utility (CI)	Attribute importance, % (SD)
**Risk of addiction**		30.6 (14.0)
High	−17.1 (−18.1 to −16.1)	
Medium	3.6 (3.3 to 3.9)	
Low	13.5 (12.6 to 14.4)	
**Amount of pain relief**		19.6 (10.5)
High	7.3 (5.4 to 9.2)	
Medium	5.0 (4.5 to 5.5)	
Low	−12.3 (−14.3 to −10.3)	
**Side effects**		13.8 (7.2)
Likely	−6.9 (−7.3 to −6.5)	
Unlikely	6.9 (6.5 to 7.3)	
**Functional independence**		12.3 (7.8)
Independent	6.2 (5.8 to 6.6)	
Dependent on others	−6.2 (−6.6 to −5.8)	
**Level of trust in provider**		12.2 (5.1)
High	6.1 (5.7 to 6.4)	
Low	−6.1 (−6.4 to −5.7)	
**Cost**		8.2 (4.0)
$0	3.2 (2.9 to 3.5)	
$10	1.9 (1.8 to 2.0)	
$25	−5.1 (−5.4 to −4.8)	
**Stigma assoc. with use**		3.3 (1.4)
Yes	−1.7 (−1.8 to −1.6)	
No	1.7 (1.6 to 1.8)	

© 2021 Baxter NB et al. *JAMA Network Open.*

eTable 22. Comparison of Responders and Nonresponders

Characteristic	Responders N	(%)	Non-responders N	(%)
**Sex**				
Female	212	(66.0)	240	(61.7)
Male	108	(33.6)	149	(38.3)
Other	1	(0.3)	0	(0.0)
**Age**				
18-19	1	(0.3)	3	(0.8)
20-29	11	(3.4)	27	(6.9)
30-39	18	(5.6)	41	(10.5)
40-49	42	(13.1)	59	(15.2)
50-59	89	(27.7)	98	(25.2)
60-69	102	(31.8)	100	(25.7)
70-79	48	(15.0)	50	(12.9)
80 or older	10	(3.1)	11	(2.8)
**Race**				
White	289	(90.0)	330	(84.8)
Black or African American	18	(5.6)	29	(7.5)
American Indian or Alaska Native	5	(1.6)	2	(0.5)
Asian	3	(0.9)	13	(3.3)
Native Hawaiian or Pacific Islander	1	(0.3)	1	(0.3)
Other	5	(1.6)	14	(3.6)
**Ethnicity**				
Hispanic	8	(2.5)	12	(3.1)
Non-Hispanic	313	(97.5)	377	(96.9)

© 2021 Baxter NB et al. *JAMA Network Open.*
